# Interactions in plasticizer mixtures used for sugar replacement

**DOI:** 10.1016/j.crfs.2023.100472

**Published:** 2023-03-07

**Authors:** R.G.M. van der Sman

**Affiliations:** a Wageningen Food Biobased Research, Wageningen University & Research, the Netherlands; b Food Process Engineering, Wageningen University & Research, the Netherlands

**Keywords:** Hygroscopicity, Polycarboxylic acids, Flory-huggins theory, Sugar replacement

## Abstract

In our quest for novel ingredients to be used in sugar replacement strategies, we have investigated the thermodynamics of polycarboxylic acids, such as citric acid. We have demonstrated the applicability of the Flory-Huggins (FH) theory to describe the thermodynamics of polycarboxylic acids solutions. Moreover, for citric acid we can describe the complete phase diagram with the theory. It shows that polycarboxylic acids have similar plasticizing and hygroscopic properties as sugars and polyols.

Regarding mixtures of polycarboxylic acids and carbohydrates, the FH theory is able to describe a) the water activity of the mixtures, b) the solubility of ternary mixtures of acids and sugars, c) the lowering of the deliquescence point for binary mixtures of crystals, and d) the melting point depression in eutectic mixtures. Unexpectingly, our investigations show there is a strong non-zero FH interaction parameter between carboxylic acids and carbohydrates. In our prior sugar replacement strategy we have assumed zero interactions between plasticizers. Here, we will readdress this assumption. Carefull investigations of solid-liquid equilibrium of eutectic mixtures involving polycarboxylic acids and/or carbohydrates, shows nearly zero interaction in eutectic mixtures consisting only of two carbohydrates or two polycarboxylic acids. We now hold the hypothesis that there is strong non-zero interaction if the mixture contains plasticizers strongly differing in the amount of hydrogen bonding groups. This strong interaction explains why these mixtures, like polycarboxylic acids and carbohydrates, are excellent candidates as deep eutectic solvents. Furthermore, we conclude that polycarboxylic acids are useful additions to the toolbox of sugar replacers, albeit that there are some limitations to their amounts used.

## Highlights

• Flory-Huggins theory applies to polycarboxylic acids.
• Complete phase diagram for citric acid.
• Organic acids can be used as coplasticizer in sugar replacement strategies.
• Strong interaction between polycarboxylic acids and carbohydrates.

## 1 Introduction

Over the last years our group has been developing theories regarding the physico-chemical properties of sugars and their replacers (van der Sman, 2013a, 2013b, 2016, 2017, 2019; van der Sman and Mauer, 2019, van der Sman, 2019). After obtaining insight regarding sugar functionality in sweet bakery products (van der Sman and Renzetti, 2018, 2020), we have been able to formulate a seemingly universal sugar replacement strategy, which has been validated for both biscuits and cakes (van der Sman et al., 2022; Renzetti and van der Sman, 2022). Our theories inform that sugar functionality, regarding food texture, is governed by two physical numbers, characterizing sugar as a plasticizer and as a humectant. These two numbers are a) the volumetric density of hydrogen bonds (available for intermolecular bonding) *φ*_*w*,*eff*_, and b) the volume averaged Flory-Huggins interaction parameter *χ*_*eff*_. Our sugar replacement strategy entails that the texture of a reference product can be mimicked, if the mixture of sugar replacers have similar values of *φ*_*w*,*eff*_ and *χ*_*eff*_ as in the reference product. We have shown that multiple solutions exist for the sugar replacement problem in biscuits, such as combinations of a small polyol and fructose oligosaccharides (FOS) (van der Sman et al., 2022). These multiple solutions provide opportunity for optimizing the reformulation for other (health) traits like dietary fiber content. To broaden the toolbox of sugar replacers we have been investigating the potential of other compounds, like (sweet) amino-acids, as sugar replacers (Van der Sman et al., 2020; Renzetti et al., 2020). For cake, we have shown indeed that combinations of amino-acids, sugars and dietary fibers (FOS) can create similar textures as in the reference product (Renzetti and van der Sman, 2022). Of course, in the presence of reducing sugars the levels of amino-acids are limited, due to the undesired color and taste formation via the Maillard reaction. In this paper,we explore further extension of our toolbox of sugar replacers with polycarboxylic acids like citric or malic acid. Similar to amino acids, the polycarboxylic acids also act as plasticizer, and as humectant. Furthermore, these acids are present in fruit preparations used in bakery products and desserts (Fügel et al., 2005), or in confectionery (Licciardello et al., 2012; Zhang and Barringer, 2018; Wang and Hartel, 2022). Hence, in sugar replacement strategies of such products, one has to account also for their plasticizing and humectant properties.

To assess their potential, we investigate the thermodynamic properties of polycarboxylic acids, as relevant to their function in sugar replacement formulations. We have collected a significant amount of research data from other scientific fields, where polycarboxylic acids play an important role, namely aerosol science (Beyer et al., 2008; Rovelli et al., 2019), phase change materials (PCM) (Ma et al., 2018), and Natural Deep Eutectic Solvent (NADES) (Dai et al., 2013; Gontrani et al., 2019; Guinet et al., 2020). For aerosols, PCM and NADES the solid-liquid equilibrium of eutectic mixtures is of particular interest. Knowledge about the thermodynamics governing this equilibrium can provides us accurate data on the interaction between plasticizers used in these eutectic mixtures. In NADES one particularly searches for mixtures with strong non-zero interactions, such that the melting point of the eutectic is starkly lowered (as compared to the ideal case) (Martins et al., 2019). In aerosols science eutectic mixtures of carbohydrates and organic acids are investigated as proxies for secondary organic aerosols, which are of importance in cloud formation (Song et al., 2016; Marsh et al., 2017, 2019). Deliquescence of such mixtures is of importance to aerosol science (Peckhaus et al., 2012), as well as food science (Salameh et al., 2006; Mauer and Taylor, 2010, van der Sman, 2017). Investigation of the solid-liquid equilibrium of eutectic mixtures will render us accurate information about the (possible) non-zero interaction between food-borne plasticizers to be used in sugar replacement. In our sugar replacement strategy we have assumed zero interaction between these sugar replacers, allowing us to characterize them with the volume-averaged Flory Huggins interaction parameter *χ*_*eff*_.

In PCM one researches binary eutectic mixtures with high latent heat and melting points in the practical range of 0 < *T* < 100^*o*^*C*. Surprisingly, promising eutectic mixtures as phase-change materials, are composed of polyols such as erythritol/xylitol mixtures (Diarce et al., 2015; Del Barrio et al., 2016; Gunasekara et al., 2018). Hence, investigations of these eutectic mixtures will provide data, for testing our assumption of zero interaction. Consequently, we also address data on eutectic mixtures composing of polyols and/or sugars.

Our interest in polycarboxylic acids also stems from one of our other investigations concerning the use of side-streams from the plant food processing industry as a potential source of sugar replacers using hydrolysis products from cell wall polysaccharides. These hydrolysis products may also contain some oligomeric organic acids, originating from pectin. Polycarboxylic acids (like citric acid or oxalic acid) can be viewed as a proxy for these hydrolysis products. We foresee the application of the organic acids in combination with sugars and/or their replacers (like polyols or hydrolyzed polysaccharides from cell wall materials). Hence, the interactions in mixtures of polycarboxylic acids and carbohydrates are also important in that respect.

In previous studies (van der Sman, 2013b, 2017) we have characterized the humectant (hygroscopic) properties of carbohydrates in terms of the Flory-Huggins-Free-Volume (FHFV) theory (van der Sman and Meinders, 2011, van der Sman, 2017). Here, we first investigate whether the thermodynamics of organic polycarboxylic acids can also be described with Flory-Huggins theory. Polycarboxylic acids are weak acids with a low amount of dissociation. Hence, it is assumed that for describing the water activity of their solutions, they can be treated as neutral solutes like carbohydrates (Fontan and Chirife, 1981). Literature shows that the glass temperature of all (dry) polycarboxylic acids, *T*_*g*,*s*_, is below room temperature. Hence, we do not have to invoke the FHFV theory, and the regular Flory-Huggins theory suffices. Furthermore, we think the relative simplicity of Flory-Huggins theory can make it a viable alternative to thermodynamic theories, In the fields of aerosol science, PCM and NADES one sees the thermodynamic theories UNIFAC and PC-SAFT more applied than FH theory (Peng et al., 2001; Held et al., 2011; Marsh et al., 2017; Pontes et al., 2017). Our choice of FH is driven by the objective to describe food materials with a single thermodynamic theory, such that we can use it in food design or reformulations. From our perspective FH theory is an obvious choice, as it has shown its applicability earlier to both small solutes like sugars and biopolymers like starch and proteins. Still, we think the relative simplicity of Flory-Huggins theory can make it a possible alternative to UNIFAC and PC-SAFT theories, which contain much more parameters than FH theory. A secondary goal of this study of organic acids is to extend the understanding of how the Flory-Huggins interaction parameter depends on the presence of functional groups, like hydroxyl and carboxyl groups, and molecular size, as initiated in our previous paper (Van Der Sman, 2019).

After characterizing the thermodynamics of polycarboxylic acids solutions, we investigate their interaction with carbohydrates, via applying Flory-Huggins to ternary mixtures, and eutectic mixtures of these acids and carbohydrates. In particular, the latter provides insight in possible non-zero between these plasticizers, which will be apparent as a non-zero Flory-Huggins interaction parameter (van der Sman, 2017). For that, we have collected data on melting points of a multitude of eutectic mixtures from the fields of NADES and PCM.

## 2 Flory-Huggins theory

The Flory-Huggins theory is originally developed to describe thermodynamics of mixtures of polymers and other polymers or their solvents, assuming they are electrical neutral. It bears large similarity with regular solution theory, but accounts of the large molecular size of polymers. In Flory-Huggins theory the monomer of the polymer or the solvent molecule is taken as the basic unit, instead of the complete molecules as in case of regular solution theory. In food science Flory-Huggins theory is commonly applied to describe melting behaviour of semi-crystalline biopolymers like starch or proteins (Doster et al., 1986; Biliaderis et al., 1986; Whittam et al., 1990; Cao and Leroy, 2005). Via combining it with Free-Volume theory, and assuming that the Flory-Huggins interaction parameter is composition dependent, we have shown that Flory-Huggins theory can be used to describe the complete thermodynamics of food biopolymers like polysaccharides and proteins (van der Sman and Meinders, 2011; Van der Sman, 2012). Moreover, the Flory-Huggins theory can equally well describe the thermodynamics of small neutral solutes in water. In a series of papers we have shown it to be applicable to carbohydrates like sugars, polyols, oligosaccharides and amino-acids van der Sman (2017); Van Der Sman (2019); Van der Sman et al. (2020).

We note, that the original Flory-Huggins theory, formulated for synthetic polymers, assumed a constant Flory-Huggins interaction parameter, independent of composition. But, to be able to account for interactions via hydrogen bonding it is required to have the composition dependency (van der Sman and Meinders, 2011), where we show this approximates the more fundamental cluster Pincus-Tanaka-deGennes theory. Many other authors used this assumption for hydrogen-bonding systems (Schuld and Wolf, 2001; Russell et al., 2015; Tian et al., 2019; Paulin et al., 2021), and even Flory and Erman had to apply it to describe hydrogel swelling (Erman and Flory, 1986). In most cases authors proposed a quadratic relation, as in our first paper (van der Sman and Meinders, 2011).

Flory-Huggins theory is formulated in terms of a free energy functional from which one derives the chemical potentials of the compounds present in the mixture. The free energy functional can be derived for any number of (neutral) compounds in the mixture.

The free energy functional consists of two parts: one describes the entropy of mixing, and the second part account for the enthalpic contribution of mixing. The latter can be measured as the heat of mixing. The enthalpic contribution is proportional to the so-called Flory-Huggins interaction parameter *χ*_*ij*_ and the product of compounds volume fractions *φ*_*i*_*φ*_*j*_. Below, we give the chemical potentials of compounds composing a ternary mixture of 2 plasticizers and water.

### 2.1 Chemical potentials for ternary aqueous mixtures

As stated above, we will also investigate ternary aqueous mixtures of carbohydrates and organic acids. Hence, the Flory-Huggins theory is given for ternary mixtures. The theory for binary solutions is easily derived by setting the volume fraction of one of the solutes to zero. The components present in the solution will be indicated by indices *s*, *g*, and *w* (for carbohydrate, the organic acids, and water respectively). The chemical potentials of these components follow (van der Sman, 2017):

(1) $$\begin{array}{lll} \mu_{\mathrm{liq,s}}/\nu_{s}RT & = & \frac{\ln\;\varphi_{s}}{\nu_{s}}-\left(1-\frac{1}{\nu_{s}}\right)\varphi_{w}+\left(\frac{1}{\nu_{s}}-\frac{1}{\nu_{g}}\right)\varphi_{g}+\\ & & +\left(\chi_{sg}\varphi_{g}+\chi_{sw}\varphi_{w}\right)\left(1-\varphi_{s}\right)-\chi_{gw}\varphi_{g}\varphi_{w} \end{array}$$

(1) $$\begin{array}{lll} \mu_{\mathrm{liq,g}}/\nu_{g}RT & = & \frac{\ln\;\varphi_{g}}{\nu_{g}}-\left(1-\frac{1}{\nu_{g}}\right)\varphi_{w}+\left(\frac{1}{\nu_{g}}-\frac{1}{\nu_{s}}\right)\varphi_{s}+\\ & & \left(\chi_{sg}\varphi_{s}+\chi_{gw}\varphi_{w}\right)\left(1-\varphi_{g}\right)-\chi_{sw}\varphi_{s}\varphi_{w} \end{array}$$

(1) $$\begin{array}{lll} \mu_{w}/RT & = & \ln\left(\varphi_{w}\right)+\left(1-\frac{1}{\nu_{s}}\right)\varphi_{s}+\left(1-\frac{1}{\nu_{g}}\right)\varphi_{g}+\\ & & \left(\chi_{sw}\varphi_{s}+\chi_{gw}\varphi_{g}\right)\left(1-\varphi_{w}\right)-\chi_{sg}\varphi_{s}\varphi_{g} \end{array}$$

Here, *ν*_*i*_ is the molar volume of solute *i* relative to water, *φ*_*i*_ is its volume fraction, and *χ*_*ij*_ are the Flory-Huggins interaction parameter between components *i* and *j*. *R* the universal gas constant, and *T* the temperature. The water activity is computed from the chemical potential of water via: *μ*_*w*_ = *RT* ln(*a*_*w*_). The volume fractions of the compounds will be computed using the densities *ρ*_*s*_ and molar weights *M*_*w*_ given in Table 1. The given densities are the values at room temperature. We neglect the temperature dependency of the densities, which are often not known for the whole temperature range. Given the large density difference between water and solutes, the error of this approximation is assumed negligible.

**Table 1:** Properties of polycarboxylic acids.

Name	*T*_*g*_ (K)	*N*_*C*_	*N*_*COOH*_	*N*_*OH*_	*M*_*w*_ (g/mol)	*ρ*_*s*_ (kg/m^3^)	*χ*_*gw*_
oxalic acid		2	2	0	90	1900	−0.540 ± 0.023
malonic acid		3	2	0	104	1630	+0.037 ± 0.016
succinic acid		4	2	0	118	1552	+0.338 ± 0.009
glutaric acid		5	2	0	132	1429	+0.529 ± 0.005
malic acid	255	4	2	1	134	1609	+0.011 ± 0.025
citric acid	284	6	3	1	192	1665	+0.126 ± 0.012
tartaric acid	289	4	2	2	150	1760	−0.134 ± 0.029

**Table 2:** Data sources for solubility of carboxylic acids with associated parameters. Δ*C*_*p*,*x*_ is fitted to solubility data.

Compound	References	*T*_*x*_ (K)	Δ*H*_*x*_ (kJ/mol)	Δ*C*_*p*,*x*_ (J/mol.K)
oxalic acid	Apelblat and Manzurola (1987)	464	18.6	−65.4 ± 2.6
malonic acid	(Apelblat and Manzurola, 1987) (Beyer et al., 2008)	409	23.1	90.0 ± 1.0
succinic acid	Apelblat and Manzurola (1987)	461	33.0	−3.5 ± 3.3
glutaric acid	(Apelblat and Manzurola, 1989) (Beyer et al., 2008)	371	20.7	−115.1 ± 7.8
malic acid	(Apelblat and Manzurola, 1987) (Beyer et al., 2008)	402	33.5	126.3 ± 1.1
citric acid	(De Kruif et al., 1982) (Apelblat and Manzurola, 1987) (Apelblat, 2014)	428	40.3	183.0 ± 3.7
tartaric acid	Apelblat and Manzurola (1987)	445	36.3	157.2 ± 0.6

### 2.2 Solid-liquid equilibrium

Accurate data for the Flory-Huggins interaction parameter between solutes, *χ*_*sg*_, can be obtained via investigation of the solid-liquid equilibrium, i.e. the coexistence of crystals and the ternary solution (van der Sman, 2017). This equilibrium can be computed from the conditions:

(2) $$\begin{array}{lll} \mu_{\mathrm{liq,s}} & = & \mu_{X,s}\\ \mu_{\mathrm{liq,g}} & = & \mu_{X,g} \end{array}$$

with *μ*_*X*,*i*_ the chemical potential crystalline form of the solute *i*. These conditions also hold for the solid-liquid equilibrium of binary solutions, assuming the absence of the other solute (*φ*_*s*_ = 0), as well as for eutectic mixtures (where water is absent: *φ*_*w*_ = 0).

The expressions for the chemical potential of these crystal are:

(3) $$\begin{array}{lll} \mu_{X,s} & = & -\Delta H_{x}\left(1-T/T_{x}\right)-\Delta C_{p,x}\left(T-T_{x}\right)-\frac{1}{2}\Delta \gamma_{p,x}\left(T^{2}-T_{x}^{2}\right)\\ & & -T\left(\Delta C_{p,x}-T_{x}\Delta \gamma_{p,x}\right)\ln\left(T_{x}/T\right) \end{array}$$

*T*_*x*_ is the melting point of the pure compound, Δ*H*_*x*_ is the latent heat of crystallization at *T* = *T*_*x*_, Δ*C*_*p*,*x*_ is the difference in specific heat between molten and solid phase at *T* = *T*_*x*_, and Δ*γ*_*x*_ descibes how this specific heat difference changes linear with temperature.

The solid-liquid equilibrium also determines the phenomenon of deliquescence, where mixtures of solute crystals dissolve if in contact with humid air of a critical relative humidity.

The Ross equation is proposed to compute this critical relative humidity (Salameh and Taylor, 2005). It states that the water activity of multicomponent solutions can be computed as follows(Chirife et al., 1985):

(4) $$\log\left(a_{w,mix}\right)=\underset{i}{\sum }\log\left(a_{w,i}^{\left(0\right)}\right)$$

$a_{w,i}^{\left(0\right)}$ is the partial water activity of component *i*, which would exist in a binary mixture of just the amount of water and solute *i*, as present in the multicomponent mixture. The Ross equation is said to be valid only if there is little interaction between solutes, i.e. *χ*_*sg*_ ≈ 0. We will compare the Flory-Huggins prediction with that of the Ross equation for the deliquescence lowering.

### 2.3 Glass transition

For some of the polycarboxylic acids, like citric acid, there is sufficient literature data available to construct the complete supplementary state diagram, which includes all phase transitions, but also the glass transition. The glass transition will be modelled with the Couchman-Karasz equation:

(5) $$T_{g}=\frac{y_{s}\Delta C_{p,s}T_{g,s}+y_{w}\Delta C_{p,w}T_{g,w}}{y_{s}\Delta C_{p,s}+y_{w}\Delta C_{p,w}}$$

with *y*_*s*_ = 1 − *y*_*w*_ the mass fraction of the solute, *T*_*g*,*s*_ and *T*_*g*,*w*_ are the glass transition temperature of the pure solute and solvent (water). Δ*C*_*p*,*i*_ is the change in specific heat at the glass transition of the pure compound. The values for water are well known, and they can be found in (van der Sman, 2013b).

## 3 Results

### 3.1 Water activity of polycarboxylic acids

We have collected data of water activity of various polycarboxylic acids from multiple sources as listed in Table S1. Properties of the investigated organic acids are listed in Table 1. We have listed *T*_*g*_ glass transition temperature (if available), *N*_*C*_ the number of carbon atoms, *N*_*COOH*_ the number of carboxyl groups, *N*_*OH*_ the number of hydroxyl groups, *M*_*w*_ the molar mass, *ρ* the mass density. Similar to polyols we expect that the Flory-Huggins interaction parameter is strongly determined by the number of functional groups versus the number of carbon atoms per molecule (Van Der Sman, 2019).

We have fitted the Flory-Huggins theory for binary solutions to the water activity data via the least squared error, using the *curve*_*fit* function of SciPy module of Python. The error in the Flory-Huggins interaction parameter *χ*_*gw*_ is estimated from the covariance matrix. The fitted interaction parameters and its error *χ*_*gw*_ are listed in Table 1.

**Fig. 1:**
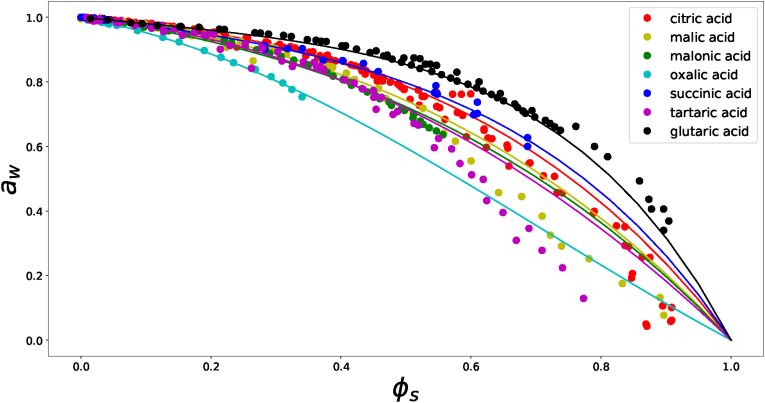
Water activity of various solutions of polycarboxylic acids at room temperature as function of solute volume fraction *φ*_*s*_, with lines the Flory-Huggins theory equation fitted to experimental data. Experimental data is from a range of literaure sources, as listed in Table S1.

The collected experimental data are shown as symbols in Fig. 1. We note that for several compounds the datasets do not cover the whole range of 0 < *φ*_*s*_ < 1 due to limited solubility of several of the polycarboxylic acids. For some compounds, experiments are performed with small aerosol droplets, which inhibit the formation of crystals because of their small size (Marsh et al., 2017). For tartaric acid, malic acid, and glutaric acid we find large deviations with theoretical prediction in the range of high acid concentrations (as measured with the aerosols). We think they deviate because the aerosol droplets were not in equilibrium with their environment at the experimental time scales, because of the low water diffusivities (Davies and Wilson, 2016; Nadler et al., 2019).

Hence, definitely within the solubility ranges of the polycarboxylic acid, the Flory-Huggins theory gives good agreement with the experimental data. Observe that some interaction parameters are nearly zero or even negative, which was also the case when we fitted Flory-Huggins theory to amino-acids (Van der Sman et al., 2020).

As in our previous paper (Van Der Sman, 2019), the results on carboxylic acids indicate that *χ*_*gw*_ depends on its hydrophobicity (as expressed by (*N*_*COOH*_ + *N*_*OH*_)/*N*_*C*_). We expect also some dependence on the size of the solute (as expressed by *N*_*C*_), but the analysed dataset on polycarboxylic acids is not conclusive in this respect.

Oxalic acid is a small molecule, and all its carbon atoms have hydrogen bonding groups, i.e. carboxyl groups. This renders a high hygroscopicity, and thus a strongly negative *χ*_*gw*_. On the other hand, glutaric acid is a large molecule with *N*_*C*_ = 5, but only two carbon atoms have hydrogen bonding groups. Consequently, glutaric acid is rather hydrophobic with a high *χ*_*gw*_. In homologues series of dicarboxylic acids (with *N*_*COOH*_ = 2 and 2 ≤ *N*_*C*_ ≤ 5) *χ*_*gw*_ increases monotonically with *N*_*C*_. Comparison of acids with *N*_*C*_ = 4 and *N*_*COOH*_ = 2 shows that an increase of *N*_*OH*_ lowers *χ*_*ws*_. These findings are in agreement with findings for polyols, where *N*_*C*_ ≥ *N*_*OH*_ (Van Der Sman, 2019).

### 3.2 Supplemented state diagram citric acid

For carbohydrates, we have concluded that the Flory-Huggins interaction parameter is independent of temperature (van der Sman and Meinders, 2011). To test whether this is the case for polycarboxylic acids, we investigate the supplemented state diagram of citric acid. Because of its importance as a food ingredient, there is a lot of data available regarding its phase behaviour, which has been collected by Apelblat (2014). The collected data include both the freezing line and boiling line, which allows the test of temperature independency of *χ*_*gw*_. At the boiling line *μ*_*w*_ = *μ*_*v*_, the chemical potential of water vapour, and at the freezing line *μ*_*w*_ = *μ*_*ice*_. Expressions for *μ*_*v*_ and *μ*_*ice*_ follow the general expression, Eq. (3), and specific expressions and parameter values can be found in (van der Sman and Meinders, 2011), where we constructed the supplemented state diagram for starch using the Flory-Huggins-Free-volume theory. The details for computing the solubility line for monohydrate crystals are given in (van der Sman, 2017), and *μ*_*w*_ is computed as described in the theoretical section.

The collected data of citric acid (Apelblat, 2014) is sufficient to construct the supplemented state diagram, which includes a) glass transition, b) freezing transition, c) boiling points, and d) solubility of monohydrate and anhydrate crystals. We compare the experimental data with the theoretical predictions of these state and phase transitions, via Couchman-Karasz and Flory-Huggins theories, assuming a constant *χ*_*gw*_.

**Fig. 2:**
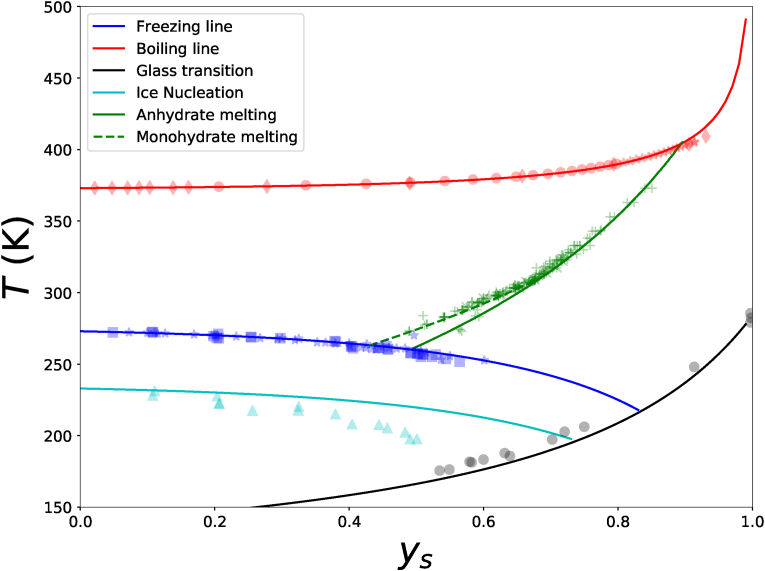
Supplemented state diagram of citric acid, showing phase transitions and glass transition as function of solute mass fraction *y*_*s*_ and temperature *T*. Experimental data (symbols) are collected by Apelblat (2014), and the solid lines are via Flory-Huggins theory. Colors of lines and symbols indicate certain phase transitions as indicated in legend. Symbols with same color, but with different shapes indicate different original data sources. (For interpretation of the references to color in this figure legend, the reader is referred to the Web version of this article.)

Results are shown in Fig. 2, where one can observe that the boiling and freezing lines are predicted well by the Flory-Huggins theory, showing the validity of the hypothesis that *χ*_*gw*_ is independent of temperature. For the glass transition we have used *T*_*g*,*s*_ = 284 K as follows from (Lu and Zografi, 1997), and Δ*C*_*p*,*s*_ = 0.425 kJ/kg/K, similar to that of carbohydrates (van der Sman and Meinders, 2011). For the solubility of anhydrate and monohydrate crystals involved fitting of multiple parameters. For the anhydrate we estimate *T*_*x*_ = 489 K, Δ*H*_*x*_ = 21 (kJ/mol), Δ*C*_*p*,*x*_ = 15 (J/mol/K), and Δ*γ*_*p*,*x*_ = 0.0 (J/mol/K^2^), and for the mono-hydrate *T*_*x*_ = 343 K, Δ*H*_*x*_ = 21 (kJ/mol), Δ*C*_*p*,*x*_ = 0 (J/mol/K), Δ*γ*_*p*,*x*_ = 0 (J/mol/K^2^). Values from literature of *T*_*x*_ and Δ*H*_*x*_ are obtained from (Crespo et al., 2018).

Similar to sucrose, the eutectic phase is not formed if the citric acid solution is frozen. The phase transition is likely kinetically inhibited via the high viscosity of the unfrozen phase of the system.

### 3.3 Solubility of other polycarboxylic acids

**Fig. 3:**
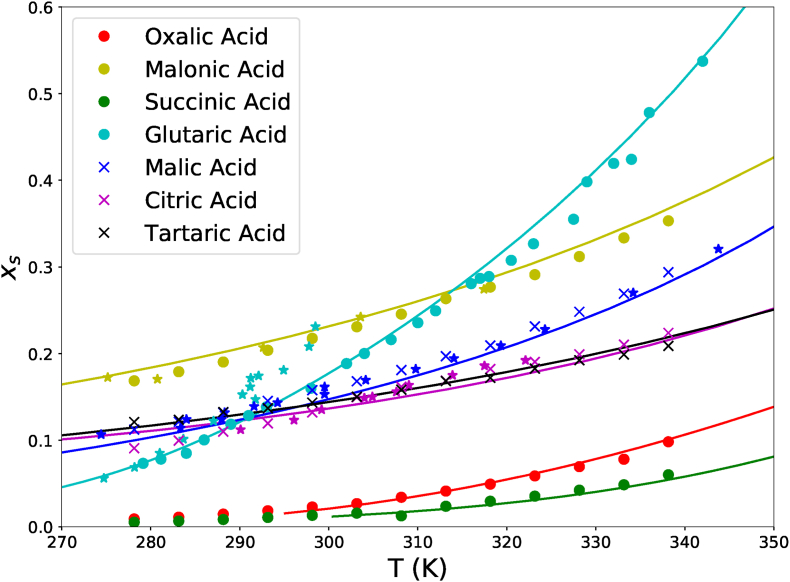
Solubility of various polycarboxylic acids as function of temperature, expressed in terms of the solute mole fraction *x*_*s*_. The used data sources are listed in Table 2. Data from Beyer et al. are indicated by star symbols.

For other carboxylic acids, we have collected data on their solubility, as listed in Table 2. The solubility data as a function of temperature is depicted in Fig. 3. We have fitted the experimental data with Flory-Huggins theory combined with Clausius-Clapeyron, Eq. (3). Values from literature of *T*_*x*_ and Δ*H*_*x*_ are obtained from (Crespo et al., 2018), which are also listed in Table 2. Furthermore, we assume that for all acids it holds Δ*γ*_*p*,*x*_ = 0. Hence, for our predictions, we have only to estimate Δ*C*_*p*,*x*_ via minimizing the error between theory and experimental data. The fitted values and their errors are listed in Table 2. The fitting is again performed with *curve*_*fit* function of Python, with errors computed from the covariance.

In Fig. 3 one can observe that after appropriate estimation of Δ*C*_*p*,*x*_ reasonable fits are obtained. We note that for low temperatures there is a deviation from the citric acid data because these data correspond to the monohydrate crystal (proper modelling of that is shown have in the state diagram 2). In our fitting, we assumed that all crystals are of the anhydrate form. From these results, we conclude that the Flory-Huggins theory can predict the solid-liquid equilibrium of polycarboxylic acids.

### 3.4 Water activity of ternary mixtures of acids and sugars

**Fig. 4:**
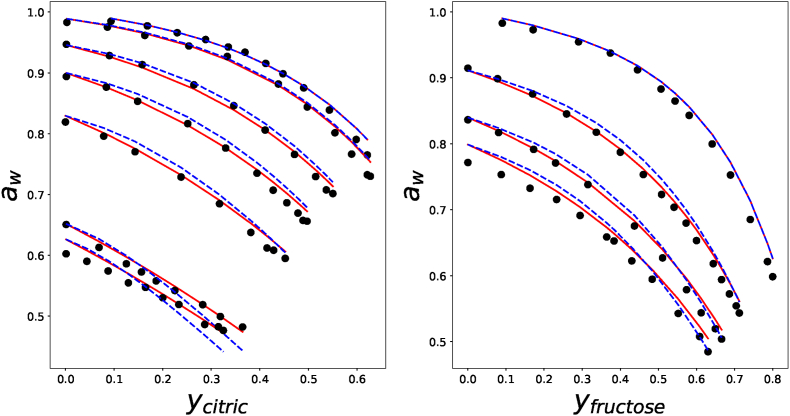
Water activity of various fructose/citric acid solutions at 25^*o*^C, with experimental data from (Kwok et al., 2010b)(symbols), and the prediction of the ternary Flory-Huggins theory with *χ*_*sg*_ = 0 (red lines) and Ross equation (blue dashed lines). In the left pane, citric acid is dissolved in fructose solutions of different molality (*m* = {0, 0.6, 3.0, 5.4, 9.1, 20.2, 22.2} from top to bottom), and in the right pane fructose is dissolved in citric acid solutions of different molality (*m* = {0, 3.8, 6.6, 8.3}). *y*_*i*_ is the mass fraction of solute *i*, based on the total mass. (For interpretation of the references to color in this figure legend, the reader is referred to the Web version of this article.)

We investigate the validity of the Flory-Huggins theory for ternary aqueous mixtures. We will also compare the theoretical predictions with the Ross equation, which assumes zero interaction between solutes. We have used experimental results of fructose and citric acid by Kwok and Mauer (Kwok et al., 2010b). Experimental results and theoretical predictions are shown in as shown in Fig. 4. The endpoints of the curves indicate the solubility limits of the ternary mixture.

Fig. 4 shows that reasonable predictions with the ternary FH theory are readily obtained if we assume *χ*_*sg*_ ≈ 0, meaning there is little interaction between the two solutes. The graph also shows that the Ross equation is indeed a good approximation of the water activity of this ternary mixture, but the Flory-Huggins theory is more accurate. The accuracy can be increased via fine-tuning *χ*_*sg*_.

**Fig. 5:**
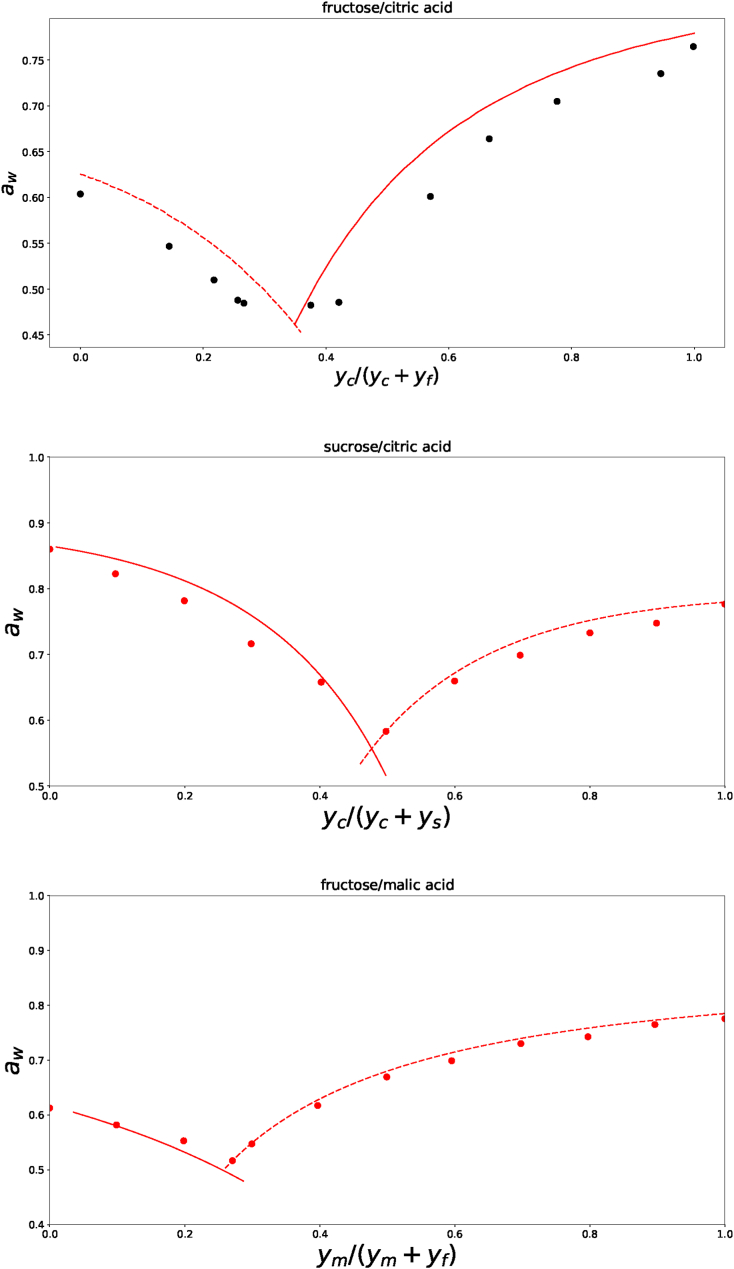
Solubility lines of the mixtures of a) anhydrous citric acid and anhydrous fructose, b) anhydrous citric acid and sucrose, and c) malic acid and anhydrous fructose at 25^*o*^*C* in the RH-state diagram. The mixture composition is represented by their mass ratio on dry matter basis. Experimental data (symbols) are from (Kwok et al., 2010b) (black symbols) and (Dupas-Langlet et al., 2017) (red symbols). The lines are calculated with the ternary Flory-Huggins theory, using the fitted values of *χ*_*sg*_. The least squares method shows *χ*_*sg*_ = −0.28 for citric acid/fructose, *χ*_*sg*_ = −0.59 for citric acid/sucrose, and *χ*_*sg*_ = −0.16 for malic acid/fructose. (For interpretation of the references to color in this figure legend, the reader is referred to the Web version of this article.)

A more accurate determination of the interaction parameter is via investigation of the solubility of the ternary mixtures. We modelled the solubility at T = 25^*o*^C of three ternary mixtures, namely solutions of a) citric acid and fructose, b) citric acid and sucrose, and c) malic acid and fructose. Experimental data is from (Kwok et al., 2010b) and (Dupas-Langlet et al., 2017). The results are shown in Fig. 5. Note, the experimental data represent two branches of solid-liquid equilibrium. The left branch presents the equilibrium between carbohydrate crystals and the molten mixture, while the right branch presents the equilibrium between polycarboxylic acid crystals and the molten mixture of the carbohydrate and acid. While fitting the theory we must make a distinction between the two branches. Via prelimenary fittings we have estimated the eutectic point, where the two branches intersect. Based on this estimate of the eutectic point, we assigned the experimental data onto either one of the two branches. Of course, the theoretical lines beyond the eutectic point do not have a physical meaning. It is an artifact of our plotting procedure.

Here, we have found that these results can only be explained by Flory-Huggins theory using a significant interaction between the acids and sugars: for citric acid and fructose *χ*_*sg*_ = −0.28 ± 0.04, for citric acid/sucrose *χ*_*sg*_ = −0.59 ± 0.04, and for malic acid/fructose *χ*_*sg*_ = −0.158 ± 0.013.

### 3.5 Deliquescence point lowering

For citric acid - fructose mixtures, we have determined the deliquescence lowering, via computing the solubility at different temperatures using the ternary FH theory, using *χ*_*sg*_ = −0.28. From the intersection of the solubility lines, as shown in Fig. S1, we obtain the composition of the eutonic point, where two crystals and the ternary solution are in equilibrium. Via the ternary Flory-Huggins theory, we can compute the water activity corresponding with the composition at the eutonic point. This water activity should equal the deliquescence point. We have compared our calculations with the deliquescence lowering measured by (Lipasek et al., 2013), and the predictions with the Ross equation. These results are listed in Table 3. As one can observe, the predictions of the FH theory are much better than that of the Ross equation, which is due to the strong interaction between citric acid and fructose, i.e. *χ*_*sg*_ = −0.28 as determined above. Hence, it strongly deviates from the underlying assumption of the Ross equation, namely the zero value of the interaction parameter, *χ*_*sg*_ = 0.

**Table 3:** Deliquescence lowering in citric acid-fructose mixtures, from experimental data (Lipasek et al., 2013) and theoretical predictions.

Temperature	Experimental RH	Prediction FH	Prediction Ross
20^*o*^C	0.489	0.500	0.510
25^*o*^C	0.448	0.452	0.487
30^*o*^C	0.418	0.423	0.463
35^*o*^C	0.379	0.394	0.437
40^*o*^C	0.351	0.363	0.410

### 3.6 Solid-liquid equilibrium in eutectic mixtures

Above, we have shown that the Flory-Huggins interaction parameter *χ*_*sg*_ for certain mixtures of polycarboxyliuc acids and carbohydrates can be strongly negative, as in the case of citric-acid/fructose, citric-acid/sucrose, and malic-acid/fructose. This is contrary to our expectation, i.e. in our sugar replacement strategy we have assumed zero interactions (i.e. *χ*_*sg*_ = 0). We will readdress our assumption, using experimental data on melting point depression of eutectic mixtures, from the fields of PCM and NADES, composed of polycarboxylic acids and/or carbohydrates (but without water!). Via comparing this data with predictions of the solid-liquid equilibrium via Flory-Huggins theory, accurate estimation of *χ*_*sg*_ can be obtained. The fitting procedure is similar as described above for computing the RH-state diagrams. In the following analysis the state diagrams are presented as melt temperatures as function of the mass fraction of one of the plasticizers in the eutectic mixture *y*_*i*_.

**Fig. 6:**
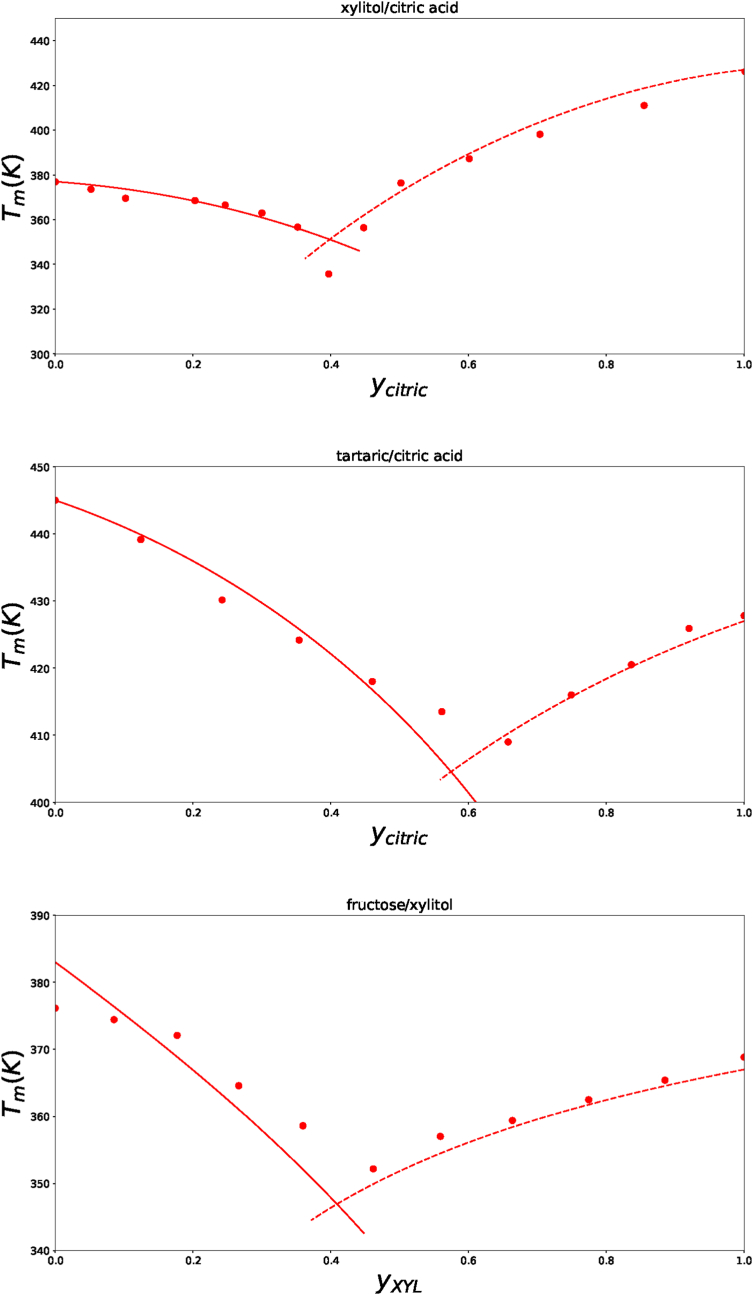
Phase diagram of the eutectic mixtures of a) citric acid and xylitol, b) citric acid and tartaric acid, c) fructose and xylitol. Experimental data (symbols) are from repsectively (Guinet et al., 2020), (Wisutthimateekorn and Lerkkasemsan, 2021) and (Meltzer and Pincu, 2012). The lines are calculated with the ternary Flory-Huggins theory, using the fitted values of *χ*_*sg*_. The least squares method shows *χ*_*sg*_ = −0.63 for xylitol/citric acid, *χ*_*sg*_ = −0.11 for tartaric/citric acid, and *χ*_*sg*_ = 0.0 for fructose/xylitol.

In field of NADES a mixture of citric acid and xylitol was investigated (Guinet et al., 2020). In Fig. 6 we show that their melting behaviour can be explained by Flory-Huggins theory. But, again, we find for a mixtures of carbohydrates and citric acids a significant interaction, with *χ*_*sg*_ = −0.63 ± 0.08. Subsequently, we investigated the interaction between carboxylic acids, namely tartaric and citric acid, using data from (Wisutthimateekorn and Lerkkasemsan, 2021). Experimental data and the theoretical prediction is shown in Fig. 6. Analysis shown that this mixture has nearly zero interaction, with *χ*_*sg*_ = −0.11 ± 0.03.

Finally, we address eutectic mixtures of carbohydrates. Parameters for *μ*_*i*,*X*_ are the same as used for the polyol solubility in water (van der Sman, 2017), or are taken from literature. The used values are listed in Table S2. In Fig. 6 we show as an example, the analysis of fructose/xylitol mixtures, with data from (Meltzer and Pincu, 2012).

The analysis of other mixtures is shown in the supplementary material. As such, in Fig. S2 we show the phase diagram of mixtures involving erythritol, xylitol and sorbitol, with data from (Diarce et al., 2015; Del Barrio et al., 2016; Gunasekara et al., 2018). In Fig. S3 we show data on eutectic mixtures of mannitol with respectively erythritol, xylitol, and sorbitol, with data collected from multiple sources by Silva et al. (2021). In Fig. S4 we show data for eutectic mixtures of arabitol and erythritol, xylitol, mannitol, or dulcitol, as studied by (Del Barrio et al., 2016). In Fig. S5 we show data of eutectic mixtures with sucrose, fructose, and glucose, as collected by (Silva et al., 2018).

Our fitting procedure shows that the Flory-Huggins theory provides good predictions for all eutectic mixtures involving carbohydrates, requiring only a small interaction parameter: |*χ*_*sg*_| ≤ 0.1.

In Table 4 we have summarized the interaction parameter values for different eutectic mixtures involving two different plasticizers, as obtained in this study and our previous studies (van der Sman, 2017, 2022). This table shows that our assumption for (nearly) zero interaction in mixtures of small carbohydrates holds true for most mixtures. The only (seemingly) exception is for sucrose/maltose mixtures. This exception is likely due to experimental difficulties. Sucrose and maltose have high solubilities, and hence the viscosity of the solution at the eutectic point is quite high. Such high viscosity also prevents crystallization of sucrose at the eutectic point during freezing (van der Sman, 2017).

**Table 4:** Interaction parameters between plasticizers.

Compound_1_	Compound_2_	Data ref.	*χ*_12_
Xylitol	Citric Acid	Guinet et al. (2020)	−0.63
Sucrose	Citric Acid	Dupas-Langlet et al. (2017)	−0.59
Fructose	Citric Acid	Kwok et al. (2010b)	−0.28
Fructose	Malic Acid	Dupas-Langlet et al. (2017)	−0.16
Tartaric acid	Citric Acid	Meltzer and Pincu (2012)	−0.11
Sucrose	Glycerol	van der Sman (2017)	0.06
Sucrose	Invert sugar	van der Sman (2017)	0.00
Sucrose	Sorbitol	van der Sman (2017)	0.08
Sucrose	Xylitol	van der Sman (2017)	0.10
Glucose	Xylitol	van der Sman (2017)	−0.05
Sucrose	Glucose	van der Sman (2017)	−0.05
Sucrose	Maltose	van der Sman (2017)	−0.22
Xylitol	Fructose	Wisutthimateekorn and Lerkkasemsan (2021)	0.00
Erythritol	Xylitol	Diarce et al. (2015)	−0.05
Erythritol	Xylitol	Del Barrio et al. (2016)	−0.05
Erythritol	Xylitol	Gunasekara et al. (2018)	−0.05
Erythritol	Sorbitol	Diarce et al. (2015)	−0.05
Xylitol	Sorbitol	Diarce et al. (2015)	−0.05
Erythritol	Mannitol	Silva et al. (2021)	0.10
Xylitol	Mannitol	Silva et al. (2021)	0.00
Sorbitol	Mannitol	Silva et al. (2021)	−0.10
Erythritol	Arabitol	Del Barrio et al. (2016)	0.12
Xylitol	Arabitol	Del Barrio et al. (2016)	−0.05
Mannitol	Arabitol	Del Barrio et al. (2016)	0.0
Dulcitol	Arabitol	Del Barrio et al. (2016)	0.0
Glucose	Fructose	Silva et al. (2018)	0.00
Glucose	Sucrose	Silva et al. (2018)	0.00
Fructose	Sucrose	Silva et al. (2018)	0.00

Concludingly, the assumption of zero interaction fails only for mixtures of polycarboxylic acids and carbohydrates, where often a strong negative interaction parameter is found. Probably, only if the type of functional groups differs strongly between two plasticizers, their interaction parameter is strongly non-ideal. The strong non-zero interaction is the common property of eutectic mixtures used for NADES (Choi et al., 2011; Dai et al., 2013). Via NMR and FTIR measurements it is shown that NADES (for example consisting of polycarboxylic acids and carbohydrates) form supramolecular aggregates, which are energetically favourable to form (Choi et al., 2011; Zheng et al., 2022; Lorenzetti et al., 2022). The supramolecular aggregates form via hydrogen bonds. It is known that hydrogen bonds work cooperatively, which can explain the strong interaction between sugars and polycarboxylic acids - both rich in hydrogen-bonding groups (as quantified with *N*_*OH*_ and *N*_*COOH*_) (El Achkar et al., 2020; Lorenzetti et al., 2022).

Other literature findings seem to confirm the above hypothesis that compounds strongly differing in functional groups show strong interactions. For example, mixtures between dicarboxylic acids also have nearly zero interaction (Alenova et al., 2020), while mixtures between choline-chloride and various polyfunctional carboxylic acids show strongly non-ideal (negative) interaction parameters (Crespo et al., 2018). From their results, we infer that for these mixtures the interaction parameter appears to scale with the number of hydrogen bonding groups per unit of volume of the carboxylic acid. An earlier study on mixtures of choline chloride and fatty acids shows that interaction is nearly zero, which can be explained by the low volumetric density of hydrogen bonds (Crespo et al., 2017). Strong non-ideal (negative) interactions are also found for mixtures of choline-chloride and carbohydrates (sugars and polyols) (Martins et al., 2019). There have been investigations of predictions of interaction parameters (using regular solution theory), but this proved to depend on quite a number of molecular properties (about 10) (Chen et al., 2022). Hence, much more data on solid-liquid equilibrium for mixtures of organic acids and carbohydrates are required for obtaining correlative relations of *χ*_*sg*_ and molecular properties.

## 4 Conclusions

In this paper, we have shown that the thermodynamics of polycarboxylic acids can be described by the simple Flory-Huggins theory, as has been done before for other food plasticizers (i.e. carbohydrates). It is shown that the interaction parameter of the organic acid and water strongly depends on the molecular size, but also on the number of functional groups capable of hydrogen bonding (hydroxyl and carboxyl groups) per carbon atom - which is in line with our previous paper on non-edible polyols (Van Der Sman, 2019).

For citric acid, the complete phase diagram can be predicted, while for other polycarboxylic acids the solubility lines can be predicted, with the fitting of only one parameter (Δ*C*_*p*,*x*_). Overall, the solubility of certain acids is quite small, limiting their use as plasticizers. This is dominated by the odd-even effect: dicarboxylic acids with even *N*_*C*_ have lower solubilities than acids with an odd number of *N*_*C*_. This odd-even effect on solubility is explained by differences in molecular packing in the solid state (Zhang et al., 2013).

Via the investigation of ternary mixtures of polycarboxylic acids, carbohydrates, and water and eutectic mixtures of polycarboxylic acids and carbohydrates we have shown that the mixture solubility is starkly enhanced via the strong non-zero interaction between the solutes. Hence, the functionality of these organic acids as plasticizers is starkly enhanced in mixtures with carbohydrates. Furthermore, their strong negative interaction makes them also highly relevant candidates for Natural Deep Eutectic Solvents (NADES) (Dai et al., 2013; Guinet et al., 2020).

All-in-all polycarboxylic acids are a valuable addition to the toolbox for sugar replacement. Similar to carbohydrates and (sweet) amino-acids, they act both as plasticizer and humectant. Of course, their levels of usage can still be limited by other factors, such as too acidic pH, or hydrolysis of sugars (Kwok et al., 2010a). But, compounds like citric acids also have other positive traits like antioxidant and antimicrobial functionality, and it can contribute to masking off-tastes of stevia (Pielak et al., 2020). If the levels of these acids are limited, the significance of the strong interaction with carbohydrates (as expressed in *χ*_*sg*_) will be limited, as the contribution to hygroscopicity scales with *χ*_*sg*_*φ*_*s*_*φ*_*g*_.

## CRediT authorship contribution statement

**R.G.M. van der Sman:** I am the sole author of the paper, and I have performed the research myself using experimental data from literature.

## Declaration of competing interest

The authors declare that they have no known competing financial interests or personal relationships that could have appeared to influence the work reported in this paper.

## Data Availability

Data will be made available on request.
