# Supplementary material for: Interactions in plasticizer mixtures used for sugar replacement
Source: Curr Res Food Sci. 2023 Mar 7;6:100472. doi: 10.1016/j.crfs.2023.100472 (PMC10024087; doi:10.1016/j.crfs.2023.100472)
Supplement: Multimedia component 1 [file mmc1.pdf]

Supplementary Material to: Interactions in plasticizer mixtures used for sugar replacement

R.G.M. van der Sman[1,2]

[1] *Wageningen Food Biobased Research*, [2] *Food Process Engineering, Wageningen University & Research, the Netherlands*

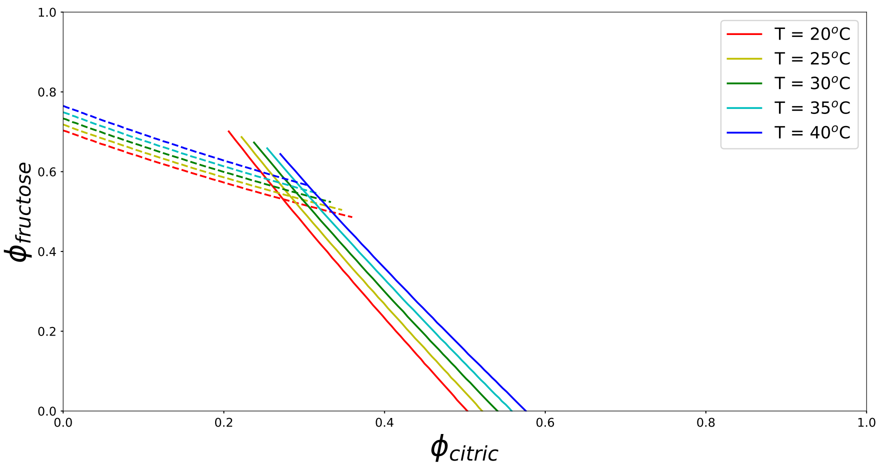

Figure S1: Solubility lines at different temperatures $20 \leq T \leq 40^o$C of mixtures anhydrous citric acid and anhydrous fructose, expressed in their volume fractions (ϕ_i).

Email address: ruud.vandersman@wur.nl (R.G.M. van der Sman[1,2])

Table S1: Data sources for water activity of polycarboxylic acids

Name	Refs
oxalic acid	(Maffia and Meirelles, 2001)<br>(Marsh et al., 2017)
malonic acid	(Marsh et al., 2017)
succinic acid	(Maffia and Meirelles, 2001)<br>(Peng et al., 2001)<br>(Marsh et al., 2017)
glutaric acid	(Peng et al., 2001)<br>(Marsh et al., 2017)
malic acid	(Velezmoro and Meirelles, 1998)<br>(Maffia and Meirelles, 2001)<br>(Peng et al., 2001)
citric acid	(Apelblat et al., 1995)<br>(Velezmoro and Meirelles, 1998)<br>(Maffia and Meirelles, 2001)<br>(Peng et al., 2001)<br>(Marsh et al., 2017)
tartaric acid	(Velezmoro and Meirelles, 1998)<br>(Maffia and Meirelles, 2001)<br>(Peng et al., 2001)<br>(Marsh et al., 2017)

Table S2: Parameters for the chemical potential of solid phase

compound	T_m (K)	Δh_m (kJ/mol)	$\Delta c_{p,X}$ (J/mol.K)	$\Delta \gamma_X$ (J/mol.K^2)	Ref
Erythritol	394	42.4	130	0.0075	(van der Sman, 2017)
Xylitol	367	38.6	175	0.012	(van der Sman, 2017)
Adonitol	375	33.5	-	-	(Carpentier et al., 2003)
L-Arabitol	374	43.2	-	-	(Carpentier et al., 2003)
	375	37.5	-	-	(Del Barrio et al., 2016)
Dulcitol	461	64.4	-	-	(Del Barrio et al., 2016)
Sorbitol	372	31.0	30	0.02	(van der Sman, 2017)
Mannitol	440	52.9	318	-0.039	(van der Sman, 2017)
Maltitol	425	52.9	230	-0.012	(van der Sman, 2017)

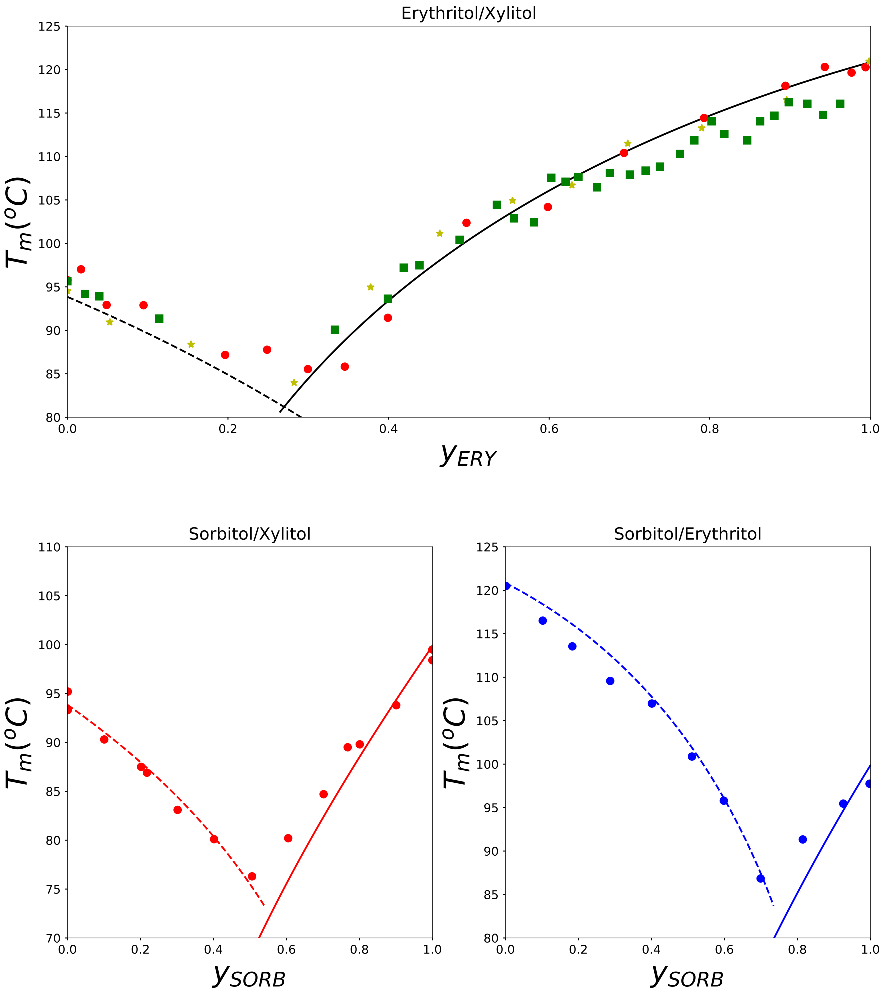

Figure S2: Phase diagram of the eutectic mixture of combinations of erytritol, xylitol and sorbitol. Experimental data (symbols) are from (Diarce et al., 2015; Del Barrio et al., 2016; Gunasekara et al., 2018). The lines are calculated with the ternary Flory-Huggins theory, using the fitted values of χ_{sg}. The least squares method shows $\chi_{sg} = -0.05$ for all mixtures.

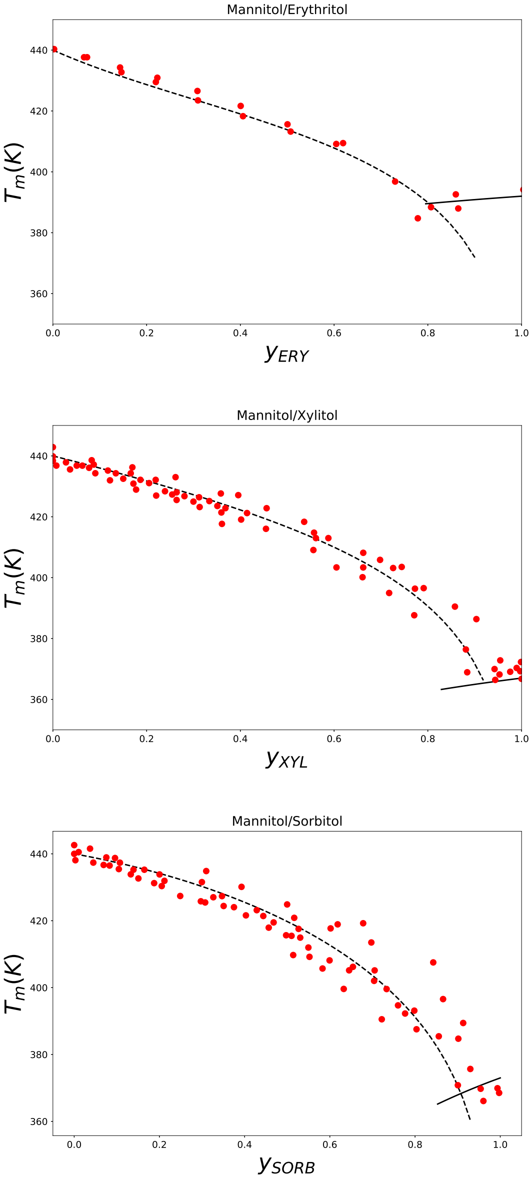

Figure S3: Phase diagram of the eutectic mixtures of mannitol with either erytritol, xylitol, or sorbitol. Experimental data (symbols) are from (Silva et al., 2021). The lines are calculated with the ternary Flory-Huggins theory, using the fitted values of χ_{sg}.

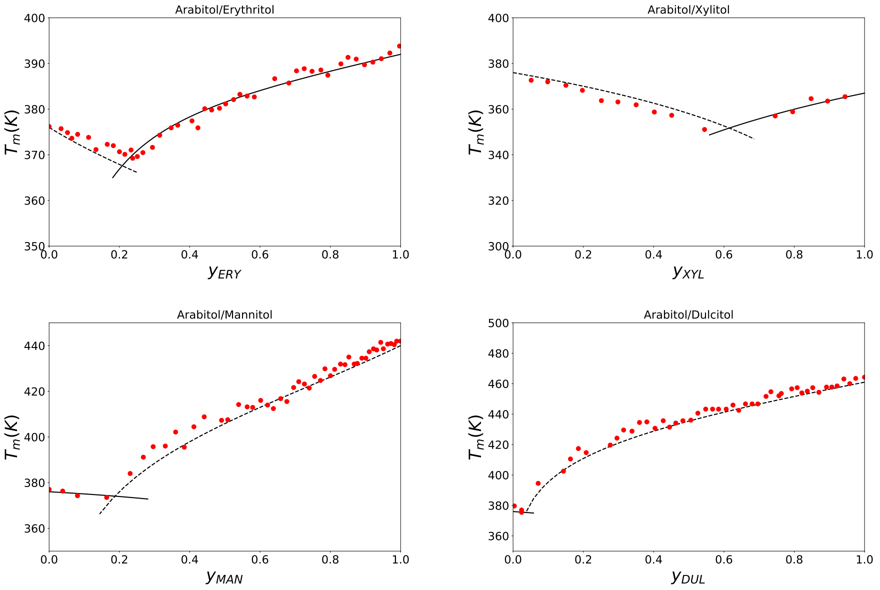

Figure S4: Phase diagram of the eutectic mixtures of L-arabitol with either erytritol, xylitol, mannitol or dulcitol. Experimental data (symbols) are from (Del Barrio et al., 2016). The lines are calculated with the ternary Flory-Huggins theory, using the fitted values of χ_{sg}.

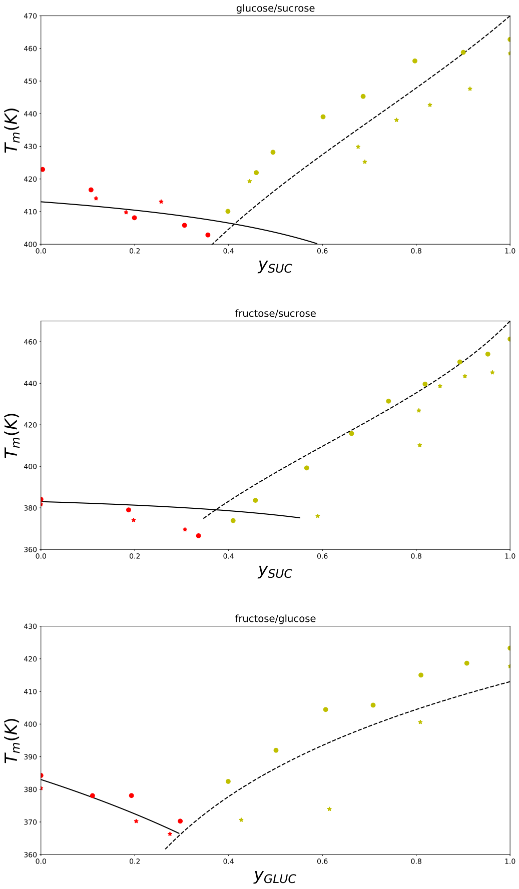

Figure S5: Phase diagram of the eutectic mixture of a) sucrose and glucose, b) sucrose and fructose, and c) glucose and fructose, with experimental data collected by (Silva et al., 2018). The lines are calculated with the ternary Flory-Huggins theory, using the fitted values of χ_{sg}.

References

Apelblat, A., Dov, M., Wisniak, J., Zabicky, J., 1995. The vapour pressure of water over saturated aqueous solutions of malic, tartaric, and citric acids, at temperatures from 288 k to 323 k. The Journal of Chemical Thermodynamics 27, 35–41.

Carpentier, L., Desprez, S., Descamps, M., 2003. Crystallization and glass properties of pentitols. Journal of thermal analysis and calorimetry 73, 577–586.

Del Barrio, E.P., Cadoret, R., Daranlot, J., Achchaq, F., 2016. New sugar alcohols mixtures for long-term thermal energy storage applications at temperatures between 70 c and 100 c. Solar Energy Materials and Solar Cells 155, 454–468.

Diarce, G., Gandarias, I., Campos-Celador, A., García-Romero, A., Griesser, U., 2015. Eutectic mixtures of sugar alcohols for thermal energy storage in the 50–90 c temperature range. Solar Energy Materials and Solar Cells 134, 215–226.

Gunasekara, S.N., Chiu, J.N., Martin, V., Hedström, P., 2018. The experimental phase diagram study of the binary polyols system erythritol-xylitol. Solar Energy Materials and Solar Cells 174, 248–262.

Maffia, M.C., Meirelles, A.J., 2001. Water activity and ph in aqueous polycarboxylic acid systems. Journal of Chemical & Engineering Data 46, 582–587.

Marsh, A., Miles, R.E., Rovelli, G., Cowling, A.G., Nandy, L., Dutcher, C.S., Reid, J.P., 2017. Influence of organic compound functionality on aerosol hygroscopicity: dicarboxylic acids, alkyl-substituents, sugars and amino acids. Atmospheric Chemistry and Physics 17, 5583–5599.

Peng, C., Chan, M.N., Chan, C.K., 2001. The hygroscopic properties of dicarboxylic and multifunctional acids: Measurements and unifac predictions. Environmental science & technology 35, 4495–4501.

Silva, L.P., Fernandez, L., Conceição, J.H., Martins, M.A., Sosa, A., Ortega, J., Pinho, S.P., Coutinho, J.A., 2018. Design and characterization of sugar-based deep eutectic solvents using conductor-like screening model for real solvents. ACS Sustainable Chemistry & Engineering 6, 10724–10734.

Silva, L.P., Martins, M.A., Abranches, D.O., Pinho, S.P., Coutinho, J.A., 2021. Solid-liquid phase behavior of eutectic solvents containing sugar alcohols. Journal of Molecular Liquids 337, 116392.

van der Sman, R., 2017. Predicting the solubility of mixtures of sugars and their replacers using the flory–huggins theory. Food & function 8, 360–371.

Velezmoro, C.E., Meirelles, A.J., 1998. Water activity in solutions containing organic acids. Drying technology 16, 1789–1805.
